# Global economic differences in modern glioblastoma care – a systematic review

**DOI:** 10.1007/s00701-026-06848-w

**Published:** 2026-03-27

**Authors:** Reuben Christopher, Livia Stauner, Mathias Spendel, Arwin Rezai, Alexander Romagna, Christoph Schwartz

**Affiliations:** 1 Department of Neurosurgery, Munich Klinik, Munich, Germany; 2 Institute of Radiation Oncology, General Hospital Klagenfurt, Klagenfurt, Austria; 3 Department of Neurosurgery, University Hospital Salzburg, Paracelsus Medical University, Salzburg, Austria; 4 Department of Neurosurgery, University Hospital Innsbruck, Medical University of Innsbruck, Anichstrasse 35, 6020 Innsbruck, Austria

**Keywords:** Costs and cost-effectiveness, Glioblastoma, Incremental cost-effectiveness ratio, Life year gained, Quality adjusted life year

## Abstract

**Purpose:**

Glioblastoma (GBM) is the most aggressive form of primary brain cancer and is associated with poor overall survival and expensive, resource intensive treatments. We performed a systematic literature review to quantify the costs and cost-effectiveness of modern glioblastoma care across global healthcare systems.

**Methods:**

A comprehensive literature search on PubMed, MEDLINE, and Cochrane databases according to the Preferred Reporting Items for Systematic Reviews and Meta-Analyses (PRISMA) guidelines using the search string (Glioblastoma OR GBM) AND (costs OR cost-effectiveness OR economic burden) identified 21 studies eligible for our research question. Costs were reviewed for all treatment modalities of the current Stupp protocol. After considering the current consumer price indexes and purchasing power parities, these were then standardized to the value of US Dollars ($) in 2024.

**Results:**

A total of 15,547 real world GBM patients were analyzed. Direct medical costs displayed extreme heterogeneity, ranging from cumulative costs of $356,481 in the United States to approximately $18,908 in India. The Stupp protocol exceeded willingness-to-pay thresholds in middle income economies. Economic evaluations of Tumor treating fields revealed Incremental Cost-Effectiveness Ratios (ICER) ranging from $862,361.37 to $940,344.39 per Life Year Gained (LYG) in France to $252,590.08 per LYG in the United States, and a more favorable per Quality Adjusted Life Year (QALY) of $45,813.91 in China.

**Conclusion:**

The cost of modern GBM treatment varies greatly between the analyzed countries with adjuvant treatment and inpatient care being the most important cost drivers in western countries in the direct medical costs analyses. Cost models show that the Stupp protocol remains a significant financial burden in resource-limited settings, underlining the cost-effectiveness of surgery in modern GBM management. There is, however, a need for uniform cost reporting to correctly assess cost-effectiveness across global healthcare systems.

## Introduction

Glioblastoma (GBM) is the most common and aggressive form of primary brain tumor in adults [2]. It is characterized by rapid infiltration of surrounding brain parenchyma, high mortality, and poor prognosis [2, 16]. Despite advances in medical care, the median overall survival remains 15–17 months after diagnosis and treatment [11]. The current standard of care, known as the Stupp protocol, consists of maximal safe surgical resection or biopsy followed by radiotherapy with concurrent and adjuvant temozolomide (TMZ) chemotherapy [12, 24, 30]. The disease requires high-end modern multidisciplinary treatment from neurosurgeons, neurooncologists, psycho-oncologists, and radiation oncologists alike [12, 24, 30]. Furthermore, modern management includes neuropathological diagnoses through molecular testing, the use of 5-aminolevulinic acid (5-ALA) to optimize surgical resection, and tumor treating fields (TTF) to improve the overall survival of such patients [6, 16, 22, 28, 29, 36].

Despite these advancements, GBM remains a resource-intensive and costly disease with over 50% of patients experiencing tumor recurrence within seven months of starting first line treatment [1, 20]. The financial burden of these modern interventions varies across the globe. While some healthcare systems may find these advanced technologies cost-effective within their specific willingness-to-pay (WTP) thresholds, others report the same interventions as economically unsustainable [2, 5, 12, 36]. While the Stupp protocol remains the gold standard for GBM treatment, an updated systematic review is required to evaluate whether new therapies are actually cost-effective. This systematic review intends to quantify the global economic differences in modern GBM care with a focus on the costs and cost-effectiveness of neurosurgery, radiotherapy, and oncological therapy regimens and the management of disease progression.

## Methods

### Search strategy and inclusion criteria

This study was performed around the PICO(T) format: in adult patients with diagnosed GBM (P), how does the implementation of modern Stupp-based care (I) differ across international healthcare systems (C) in terms of quantifiable direct medical costs and cost-effectiveness (O) in patients treated from 2005 onwards i.e. since the introduction of the Stupp protocol (T)? A comprehensive literature search was conducted in the PubMed, Medline, and Cochrane Library databases until the 1 st of December 2025 [5, 23, 32]. The search strategy utilized a combination of Medical Subject Headings (MeSH) and Boolean operators. The full search string applied was: (Glioblastoma OR GBM) AND (costs OR cost-effectiveness OR economic burden). The reference lists of the included studies were searched to include more studies. The search was performed without language restrictions according to PRISMA-S guidelines [25].

Only studies with quantifiable costs or cost-effectiveness data were included. Papers with cost models were included. Case reports and animal experiments were excluded. Studies which included a treatment period prior to 2005 were excluded. Non-English articles were included in the search but if the papers did not include an English version they were omitted from this review.

### Data extraction

All data was extracted from the text, tables, figures, and supplementary material of the presented studies. Two reviewers (R.C. and L.S.) independently screened the titles and the abstracts. Disagreements were resolved by structured discussion until consensus was reached. A third reviewer (C.S.) was consulted when consensus could not be reached.

Data on direct and indirect costs, including costs of individual therapeutic modalities, were collected. Cost-effectiveness data including incremental cost-effectiveness ratio (ICER), incremental cost-utility ratio (ICUR), life years gained (LYG), quality adjusted life year (QALY), quality adjusted life month (QALM) data, willingness to pay (WTP), net monetary benefit (NMB) and net health benefit (NHB) were collected. In economic studies Cost-effectiveness is evaluated using the ICER, which measures the additional financial cost required to generate one QALY. An intervention is considered economically viable only if its ICER falls below the WTP threshold. This represents the maximum amount a healthcare system would be willing to spend for that additional QALY [4, 13, 18, 33].

The ROBINS-I tool was used to assess the risk of bias across all retrospective studies and the prospective studies were evaluated according to RoB-2 tool [26, 27]. The cost models were evaluated using the 10-point Drummond criteria [8].

### Standardization of costs

In order to homogenize results across studies, all costs were standardized as per the value of US Dollars (USD; $) in 2024. For each study, the original price base year was identified from the reported methodology. In instances where a specific cost year was not explicitly stated, the final year of the primary data collection period was utilized as a proxy to ensure that the values reflected contemporary economic conditions captured in the dataset. Based on previously published literature a two-step conversion process was applied to account for temporal and geographic disparities [6, 11, 36].

Reported costs were first inflated from their original base year to 2024 levels using the national Consumer Price Index (CPI) specific to the country of the study. These inflation-adjusted values were then converted from their local currencies into USD using 2024 Purchasing Power Parity (PPP) rates. These values were obtained from the World Bank [34]. The final standardized cost was calculated across studies using the following formula:

$${\text{Cost in USD}}_{2024}={\mathrm{Cost}}_{\mathrm{original}}\times \left(\frac{{\mathrm{CPI}}_{2024}}{{\mathrm{CPI}}_{\mathrm{original}}}\right)\times \left(\frac{1}{{\mathrm{PPP}}_{\mathrm{Local},2024}}\right)$$

### Survival estimation and transition probabilities

As mentioned in several studies, monthly transition probabilities between the state of health of patients were estimated using the Declining Exponential Approximation of Life Expectancies (DEALE) method [2, 18]. This approach assumes that survival follows a decreasing exponential curve characterized by a constant hazard of death. Since clinical trial data typically reports results as median values, overall survival (life expectancy) was derived by applying a 1.44 multiplier to the reported median survival (Mean = Median/ln(2)). The monthly transition rate (*r*) was calculated using the relationship: r =  − ln (0.5)/median survival, which was subsequently converted into cycle-specific probabilities (*P*) using the formula: *P* = 1 − *e*^−*r*^. This constant hazard assumption is commonly used in GBM economics due to the characteristically short life expectancy of such patient populations [4, 33].

## Results

### Literature search

**Fig. 1:**
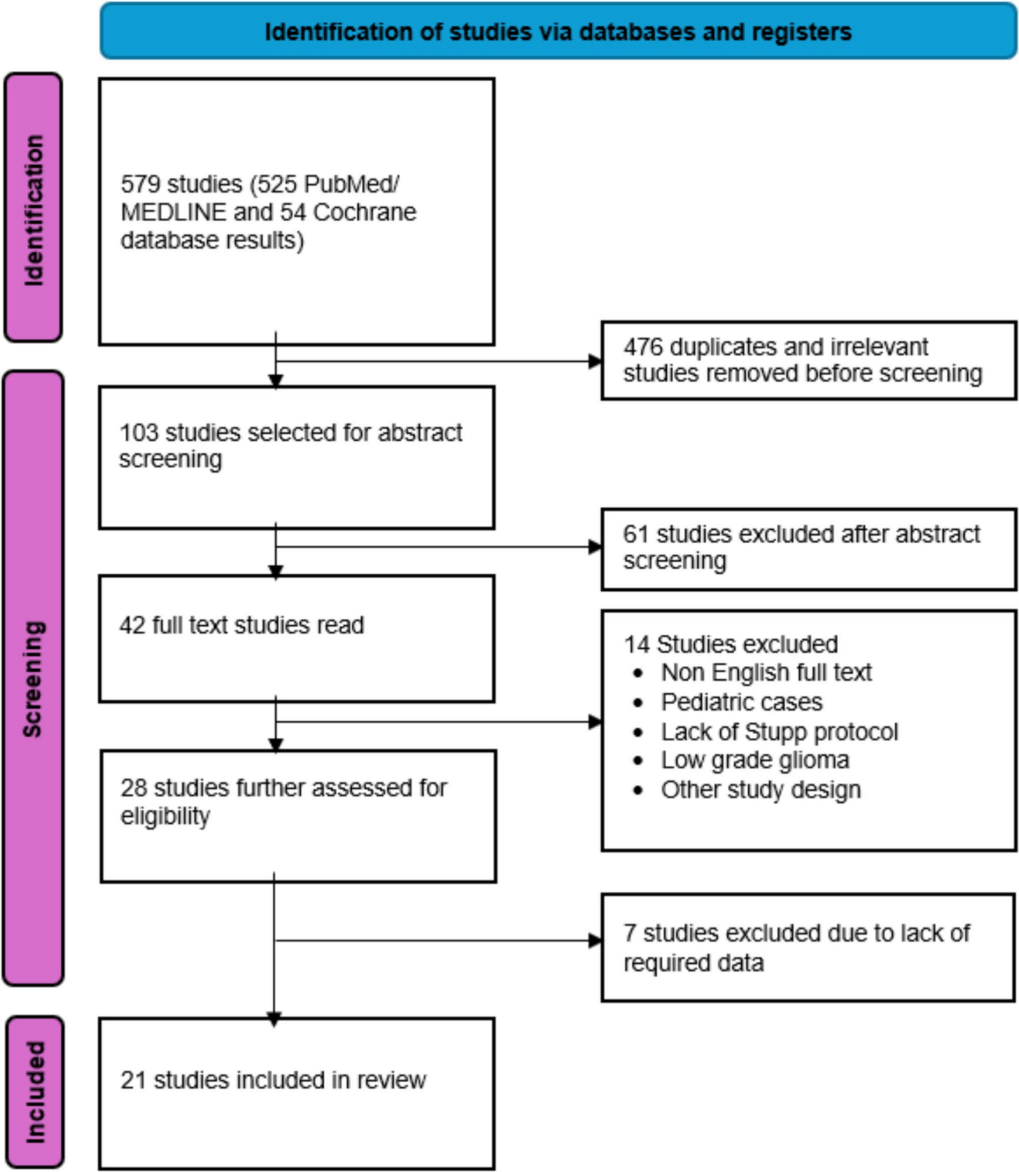
Flow diagram of literature search in accordance with PRISMA guidelines

The full results of the literature search are presented in Fig. 1. The electronic database search yielded a total of 579 studies. After the removal of 476 duplicates and studies with irrelevant titles, 103 abstracts were screened and 61 were excluded. Forty-two full text articles were read thoroughly, from which 21 studies met the requirements of our research question and had the required data. These included ten studies with direct costs of which eight were retrospective, one prospective study, and one prospective trial based evaluation. The remaining eleven were cost models of which seven were Markov models, three were partitioned survival models and one was a decision analytic model. Table 1 depicts a summary of the included studies.

**Table 1:** Basic study demographics

Study Name	Year	Country	Cost Year	Currency Reported	Level Of Evidence	Type of Study	Number Of GBM Patients	Outcomes Evaluated	Conclusion
Aly et al	2020	USA	2007–2013	USD	4	Retrospective observational	4,308	OS, resource use, and direct medical costs	Poor survival and excessive costs highlight the need for novel interventions
Bernard-Arnoux et al	2016	France	2014	EUR	2	3-health-state Markov model	MM	Incremental LYG and ICER	TTF ICER is far beyond thresholds due to device cost
Chen et al	2024	China	2024	CYN, USD	2	Markov model simulation	MM	ICER, QALY and total costs	BEV + LOM is not a cost-effective first-line option for Chinese payers
Connock et al	2019	France	2017	EUR	2	Partitioned survival model	PSM	LYG, extra cost, and ICER	TTF remains not cost-effective, limiting its use
DeWitt et al	2017	USA	2015	USD	4	Retrospective cohort analysis	431	IDH detection rate and cost savings	Age-based sequencing cutoffs result in significant cost and time savings
Guzauskas et al	2019	USA	2017	USD	2	Partitioned survival model	PSM	LY, QALY, and ICER	TTF increases lifetime survival and is cost-effective in the US
Gupta et al	2021	India	2019	INR, USD	2	Markov model analysis	MM	LY, QALY, and ICER	TMZ is not cost-effective for GBM in India at current prices
Henaine et al	2016	France	2014	EUR	4	Pharmacoepidemiologic study	217	OS, patterns of care, and total direct cost	OS improved over time, but total cost of care also increased
Jiang et al	2017	USA	2014	USD	4	Retrospective claims analysis	2,921	PPPM and cumulative costs	Direct medical costs of newly diagnosed GBM are substantial in the US
Lamers et al	2008	Netherlands	2004	EUR, CHF, CAN	2	Phase III Trial-based economic evaluation	219	Restricted mean survival and ICER	ICER for TMZ is comparable to accepted first-line cancer chemotherapies
Messali et al	2013	USA	2011	USD	2	Cost-utility Markov model	MM	QALY gained and ICER	Both branded and generic TMZ are cost-effective in the US context
Motomura et al	2024	Japan	2022	JPY	4	Questionnaire survey	733	Initial therapy costs and monthly cost	GBM treatment is expensive, and cost ineffective therapies like BEV are frequently used
Norden et al	2019	USA	2016	USD	4	Retrospective claims analysis	4,071	HCRU, costs, and patterns of care	Treatment costs were substantial and HCRU burden was unmet
Panje et al	2019	Switzerland	2017	CHF	2	Decision analytic model	DM	Mean treatment costs per patient	Institutional algorithms show high variability in treatment costs
Picart et al	2024	France	2021	EUR	1	Randomized phase III trial	147	GTR rate, PFS, OS, and stay costs	5-ALA FGS is a cost-effective tool that safely optimizes resection
Ray et al	2014	USA	2006–2010	USD	4	Population-based study	2,272	Median survival and post-index costs	TMZ significantly increases cost and potentially indicates greater disease severity
Slof et al	2015	Spain	2013	EUR	4	Retrospective observational	228	Incremental cost per CR and QALY	5-ALA FGS entails moderate cost increases and is a cost-effective innovation
Thakar et al	2025	India	2024	INR, USD	4	Retrospective generalized CEA	MM	Societal costs, QALYs, and ICER	Surgery alone is cost-effective in India, whereas the Stupp protocol is not
Waschke et al	2018	Germany	2015	EUR	2	Time-dependent Markov model	MM	QALY and ICER	Open-ended TMZ is effective but expensive; ICER is comparable to standard care
Wu et al	2012	China	2011	CNY, USD	2	Subgroup economic Markov model	MM	Costs, QALY, and subgroup ICERs	TMZ is not a cost-effective option for GBM in resource-limited settings
Xiang et al	2024	China	2022	CNY, USD	2	Partitioned survival model	PSM	Expected costs, QALYs, and ICER	Incorporating TTF into TMZ treatment is cost-effective in China

### Demographics

A total of 15,547 real-world GBM patients were analyzed. The patients included in model studies were excluded from the sample size calculation. Ten studies focused on direct medical costs, resource utilization, and descriptive financial burdens. Eleven studies used Markov or Partitioned Survival models to calculate ICERs per QALY or LYG. Eight studies (38.1%) were based in Europe, seven (33.3%) in North America and the remaining six (28.6%) were from Asia.

### Risk of bias assessments

**Fig. 2:**
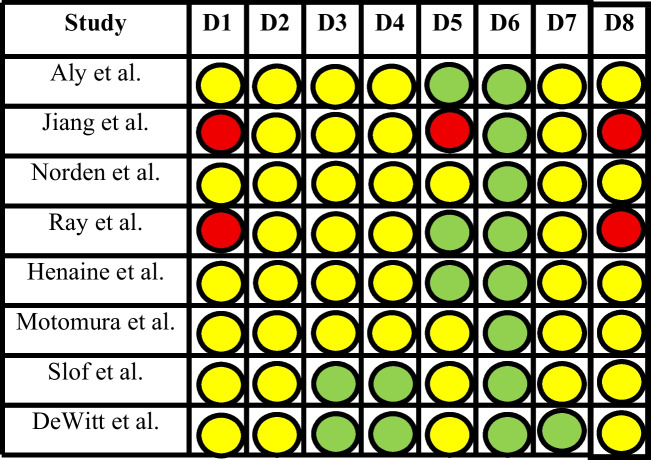
Risk of bias assessment of the retrospective cohorts using the ROBINS-I Tool (D1 – D8: Biases due to confounding, selection of participants, classification of interventions, deviation from intended interventions, missing data, measurement of outcomes, selection of reported results and overall bias, respectively)

**Fig. 3:**
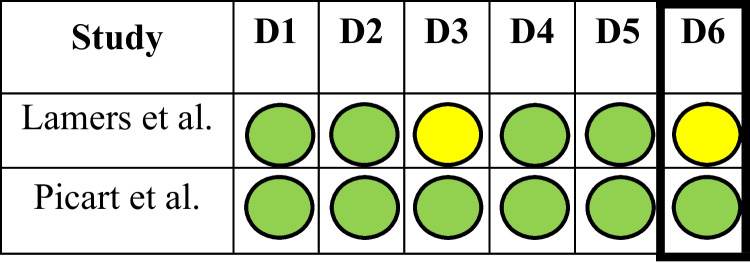
RoB-2 tool to assess risk of bias for prospective cohorts (D1 – D6: Biases due to randomization, deviations from intended interventions, missing outcome data, measurement of outcomes, selection of results and overall bias, respectively)

**Fig. 4:**
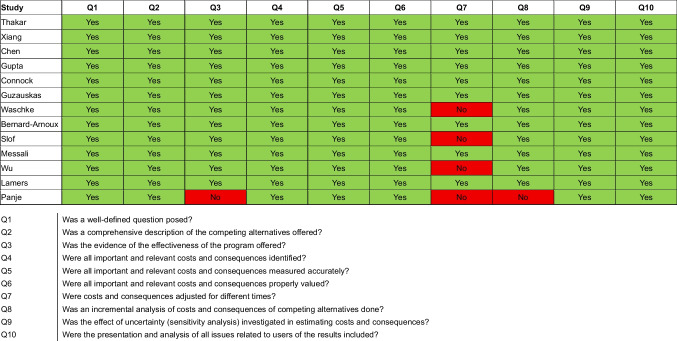
Drummond’s criteria for the assessment of economic evaluation studies

Most retrospective studies showed "moderate" risk of bias due to confounding variables inherent in non-randomized claims data. The “high” risk stratification of Jiang et al. and Ray et al. was due to patient selection protocols that excluded those not receiving specific therapies, potentially inflating survival, and cost estimates. The randomized control trial of Picart et al. showed the highest quality evidence for the cost effectiveness of 5-ALA fluorescence-guided surgery. Adherence to the Drummond criteria was generally high (Figs. 2, 3 and 4).

### Cost reporting

After the standardization of costs (USD 2024) for the included studies, global disparities in GBM therapy were reported based on empirical direct medical costs and economic models.

#### Empirical direct medical costs

The USA showed total treatment costs up to a maximum of $356,481.23 ($17,682.35 Per Patient Per Month, PPPM) in a commercially insured patient [16]. Inpatient care and radiotherapy were observed as the primary cost drivers in the US system, often accounting for up to 60% of total expenditure [16, 24]. In terms of costs per treatment modality, Aly et al. reported a mean cost of $7,461 per craniotomy in the USA [1]. Estimates from France showed that the cost of GBM surgery ranged from $13,313.09 to $15,993 per craniotomy [15]. Japan showed craniotomy costs of $13,200 [19]. French studies showed direct costs of $15,993 for primary surgery [27]. The isolated cost of the adjuvant radiochemotherapy with TMZ in the US was $137,203.2 [24]. European cohorts reported PPPM costs of $4,197 for adjuvant TMZ alone [15]. Motomura et al. reported a high fixed cost for next-generation sequencing (NGS) panels at $5,600 in Japan compared to Dewitt et al.’s $2,376 in the USA [7, 19]. See Tables 2 and 3 for the full standardized calculations of direct costs.

**Table 2:** Costs of primary surgery and surgery for recurrence/progression

**1. Primary Surgery**			
**a. Direct Medical Costs**			
**Study**	**Year**	**Country**	**Cost ($)**
Aly et al	2020	USA	7,461
Henaine et al	2016	France	15,993
Motomura et al	2024	Japan	13,200
**b. Economic Models**			
**Study**	**Year**	**Country**	**Cost ($)**
Picart et al	2024	France	13,313.09
Wu et al	2012	China	12,051
Thakar et al	2025	India	2,587.5
**2. Surgery for Recurrence/Progression**			
**a. Direct Medical Costs**			
**Study**	**Year**	**Country**	**Cost ($)**
Henaine et al	2016	France	19,258.02
**b. Economic Models**			
**Study**	**Year**	**Country**	**Cost ($)**
Panje et al	2019	Switzerland	27,840
Lamers et al	2008	Switzerland	8,400
Lamers et al	2008	Netherlands	4,513.52
Xiang et al	2024	China	3,254.09

**Table 3:** Costs of different GBM treatment modalities (Total costs, costs of RT + concomitant TMZ + adjuvant TMZ, cost of adjuvant TMZ alone, cost of RT alone, ICERs of adjunctive therapies)

**1. Total longitudinal costs of GBM treatment (diagnosis to death)**						
**a. Direct Medical Costs**						
**Study**	**Country**	**Time Horizon**	**Stated OS (Months)**	**Standardized Mean OS(DEALE)**	**Total Cost($)**	**Costs PPPM ($)**
Norden et al	USA	12 Months	11.4 (follow up)	20.2	212,940.5	12,969
Jiang et al	USA	5 Years	14 (follow up)	20.5	356,481.23	17,682.35
Ray et al	USA	12 Months	14.2 (median)	16.4	256,114.08	12,962.88
Aly et al	USA	Lifetime	8.8 (mean)	8.8	167, 586.3	15,148.35
Henaine et al	France	Lifetime	17.5 (median)	25.2	133,987.05	5,316.39
Motomura et al	Japan	12 Months	14.6 (median)	21	93,000	3,568.03
**b. Economic Models**						
**Study**	**Country**	**Time Horizon**	**Stated OS (Months)**	**Standardized Mean OS(DEALE)**	**Total Cost($)**	**Costs PPPM ($)**
Wu et al	China	5 Y	14.6 (median)	21	76,195.08	3,624.66
Thakar et al	India	15 Months	14.5 (median)	20.9	18,908.6	905.15
**2. Costs of RT + concomitant TMZ + adjuvant TMZ**						
**a. Direct Medical Costs**						
**Study**	**Country**	**Time Horizon**	**Median OS (Months)**	**DEALE OS (Months)**	**Total Cost ($)**	**Cost in PPPM ($)**
Ray et al	USA	12 Months	14.2	20.5	137,203.2	11,433.6
Aly et al	USA	Lifetime	8.8	12.7	99,989.1	7,892.1
Jiang et al	USA	5 Years	-	14.0	131,997.18	9,428.37
Norden et al	USA	12 Months	14.6	21.02	110,778.84	5,270.13
Henaine et al	France	Lifetime	17.5	17.5	34,133.31	1,951.11
Motomura et al	Japan	6 Years	-	21.02	9,000	1,500
**b. Economic Models**						
**Study**	**Country**	**Time Horizon**	**Median OS (Months)**	**DEALE OS (Months)**	**Total Cost ($)**	**Cost in PPPM ($)**
Messali et al	USA	5 Years	14.6	21.02	24,726.71	1,175.94
Lamers et al	Netherlands	2.5 Years	14.6	15.12	34,382	2,274.6
Wu et al	China	5 Years	14.6	21.02	66,287.52	3,154.32
Thakar et al	India	Lifetime	14.5	20.89	9,482.6	453.95
Gupta et al	India	Lifetime	14.6	22.2	6,658.89	299.95
**3. Costs of adjuvant TMZ alone**						
**a. Direct Medical Costs**						
**Study**	**Country**	**Duration**	**OS (Mean or DEALE, Months)**		**Total Maintenance Cost ($)**	**Cost in PPPM ($)**
Henaine et al	France	6 Months	17.5		15,174.54	2,529.09
**b. Economic Models**						
**Study**	**Country**	**Duration**	**OS (Mean or DEALE, Months)**		**Total Maintenance Cost ($)**	**Cost in PPPM ($)**
Guzauskas et al	USA	6 Months	25.08		25,454.08	4,241.92
Messali et al	USA	6 Months	21.02		16,325.55	2,721.62
Waschke et al	Germany	5 Cycles	21.02		20,988	4,197.6
Wu et al	China	6 Months	21.02		37,674	6,278.22
Gupta et al	India	6 Months	22.2		621.18	103.53
**4. Costs of RT alone**						
**a. Direct Medical Costs**						
**Study**	**Country**	**Horizon**	**Original Median OS (Months)**	**Mean OS (DEALE, Months)**	**Total Cost ($)**	**Cost in PPPM ($)**
Aly et al	USA	Lifetime	3.6	5.18	106,662.15	23,166
Ray et al	USA	12 Months	13.6	19.58	189,891.36	15,824.16
**b. Economic Models**						
**Study**	**Country**	**Horizon**	**Original Median OS (Months)**	**Mean OS (DEALE, Months)**	**Total Cost ($)**	**Cost in PPPM ($)**
Messali et al	USA	5 Years	12.1	17.42	60,502.53	3,473.61
Lamers et al	Netherlands	2.5 Years	12.1	12.12	40,099.86	3,307.09
Wu et al	China	5 Years	12.1	17.42	16,927.56	971.1
Gupta et al	India	Lifetime	12.1	15.12	7,385.14	489.08
**5. ICERs and Incremental Costs of Adjunctive Therapies in Economic Models**						
**Study**	**Country**	**Adjunct**	**OS Benefit (Months)**	**Cost ($)**	**ICER (per QALY/LYG) ($)**	
Guzauskas et al	USA	TTF	15.0	241,455.36	252,590.08	
Xiang et al	China	TTF	29.6	112,904.54	45,813.91	
Connock et al	France	TTF	7.2	437,194.55	862,361.37	
Bernard-Arnoux et al	France	TTF	4.1	317,163.96	940,344.39	
Picart et al	France	5-ALA	NA	4,398.52	NA	
Slof et al	Spain	5-ALA	1.5	2,060.40	18,402.84	
Chen et al	China	Bevacizumab	0.5	43,612.71	174,565.03	
Wu et al	China	TMZ vs RT	3.5	59,267.52	31,658.4	

#### Economic models and cost-effectiveness

Using a Markov Model Thakar et al. reported a standardized lifetime cost of $18,908 ($905.15 PPPM) in India [31]. China showed costs of $12,051 per craniotomy, whereas India reported surgical costs of $2,587.5 [31, 35]. European studies showed that surgery for recurrence/progression proved to be more expensive, reaching a high of $27,840, whereas Chinese studies revealed lower costs in this area ($3,254.09) [21, 36]. The isolated cost of the adjuvant radiochemotherapy with TMZ was $66,287 in China [35]. European cohorts reported PPPM costs of $2,529 for adjuvant TMZ alone [33].

In France, TTFs showed ICERs ranging from $862,361.37 to $940,344.39 per LYG [2, 7]. Guzauskas et al. reported an ICER of $252,590.08 per LYG in the US context [13]. Xiang et al. reported the most favorable ICER at $45,813.91/QALY in China [36]. Fluorescence-guided surgery with 5-ALA was reported to have an ICER of $18,402.84/QALY. A French randomized phase III trial showed that 5-ALA generates significant costs of $4,398.52 per surgery [22]. In China, Chen et al. calculated an ICER of $174,565.03/QALY for Bevacizumab, far exceeding the local WTP threshold [4]. See Tables 2 and 3 for the complete summary of the economic evaluations.

## Discussion

The Stupp protocol is widely accepted as the gold standard in modern GBM care [30]. Although this was established 20 years ago, the lack of uniform and detailed cost analyses of the current treatment paradigm is alarming. This study highlights the economic challenges faced when delivering high resource intensive therapies across various global healthcare systems.

Direct medical costs showed that the total cost of GBM care showed global variations with a maximum of $356,481 in the USA. This is in accordance with current published literature [11]. We found that this disparity in our study to be driven by the intensity of inpatient hospitalization and radiation therapy. Also, the USA rely to a very high degree on commercial insurance models and intensive resource use, which create a markedly financial burden. This is significantly higher than that of other European high-income systems like France ($133,987), despite similar survival outcomes. The Asian cohort showed comparable results with increasing costs in high-income countries like Japan. Previous studies showed that the direct cost of primary craniotomy was highest in the United States (mean $10,042) [11]. We found comparable costs across France and Japan. Surgery for recurrence/progression was generally more expensive in Western cohorts.

The economic models showed lower results in developing countries like India. Thakar et al. showed costs of $2,587 per GBM resection in the Indian healthcare sector. This is in accordance with previously published literature [3, 31]. France and China showed costs ranging from $13,313 to $12,051 per craniotomy. Xiang et al. showed lower costs for surgery for recurrence/progression than the cost of primary GBM resection [36]. Modern neurosurgery relies on various neurosurgical technologies to achieve Gross Total Resection (GTR) while preserving neurological function [9]. Although this is not routinely used, 5-ALA fluorescence consistently appears as a cost-effective tool, with low ICERs without compromising on patient care. Middle income countries like China and India consistently point out that the implementation of TMZ in such healthcare systems is economically challenging [31, 35]. Notably, maximal safe resection was identified as the only cost-effective strategy (ICER $3,089/QALY) compared to no active treatment, as the full Stupp protocol exceeded local WTP threshold in most parts of India [31]. This may be due to costs associated with TMZ, repeat MRIs, and repeat surgeries. Even low cost generic variations of TMZ are unable to solve the affordability crisis. Gupta et al. estimated that a further 90% cost reduction of the generic variant would be needed to make it cost-effective [12]. Conversely, generic TMZ has drastically reduced costs in the American healthcare sector [18]. Despite being affordable in most high-income countries, financial burden remains a significant barrier against adherence to therapeutic standards.

Adjunctive therapies like BEV and TTF are not globally adopted as first-line treatments due to a lack of overall survival benefit and prohibitive costs, respectively [4]. While economic models in the USA and China demonstrate its cost-effectiveness, Japanese and European evaluations find that it exceeds WTP thresholds with ICERs ranging from $862,361 to 940,344/QALY. This disparity may be influenced by methodological differences of how the survival data was extrapolated (0.34 LYG in France vs. 2.46 QALYs in China). Furthermore, an essential intervention in high-income countries could be considered catastrophic expenditure in developing economies due to varying WTP thresholds.

Future cost models should use real-world evidence like expenses for frail and elderly populations and treatment discontinuations. Country-specific utility values should be included from a societal perspective to address financial toxicities which are currently overlooked in most cost models. Affordability and price control of standards of care need to be addressed by local healthcare authorities.

### Limitations

Although this review was not pre-registered, the methodology was not altered based on the results, limiting its reporting bias. A major limitation is the omission of other large academic databases that could have been used for the literature search. There is a lack of data regarding GBM treatment across different economic systems. Although the Stupp protocol has been the standard of care for over two decades, most studies have based their cost analyses on older studies with outdated treatment protocols. The change in diagnostic criteria for GBM since the 2021 CNS WHO classification based on IDH status and the resulting heterogeneity of survival data prior to 2021 represent a potential source of bias [17]. Because IDH mutations predict significantly improved survival, the historical survival curves used in the Markov and partitioned survival models included in this study may overestimate the true life expectancy of a true GBM, IDH- wildtype patient. As a result, the modeled lifetime costs and the projected QALYs reported in this review may be slightly inflated compared to actual clinical reality. Furthermore, the increasing therapy stratification based on O6-methylguanine-DNA methyltransferase gene (MGMT) status since 2005 may have improved the cost-effectiveness of TMZ [14]. The majority of the acquired cost data for this review was from high-income countries. Although traditional 6—week RT emerges as a primary cost driver in our analysis, contemporary neuro-oncology increasingly utilizes cost-saving hypofractionated regimens for elderly or frail patients. This would significantly diminish the economic burden of RT [10]. Another limitation is the lack of homogenized data across different healthcare systems, which may be due to intrinsic variations in commercial and in public healthcare sectors across complex economies. Furthermore, the heterogeneity of financial data poses challenges for meta-analyses. There was a lack of uniform reporting of economic data. Cost models are highly heterogeneous and are based on the results of non-indigenous healthcare systems. We resorted to using the DEALE method due to the lack of mean OS data in most retrospective studies and cost models. While mathematically convenient, the DEALE method may misrepresent actual survival curves and distort the original ICERs calculated by the primary authors. The currency standardization in USD used in this research was based on previously published literature. However, healthcare services are not fully tradable goods, and PPP conversions may distort comparisons by removing WTP thresholds from their local economic context. The reliance on administrative claims data is a limitation as it lacks relevant clinical detail. This excludes untreated patients, which in turn could potentially underestimate the total economic burden of the disease. The majority of studies used for this review adopted a healthcare payer perspective, thereby excluding indirect societal costs such as loss of productivity and caregiver burden. This potentially underestimates the true financial toxicity and the financial burden borne by GBM patients and their families. Future studies should consider treatment models in middle- and low-income countries.

## Conclusion

The cost of modern GBM treatment varies greatly between the analyzed countries with adjuvant treatment and inpatient care being the most important cost drivers in western countries in the direct medical costs analyses. Cost models show that the Stupp protocol remains a significant financial burden in resource-limited settings, underlining the cost-effectiveness of surgery in modern glioblastoma management. There is, however, a need for uniform cost reporting to correctly assess cost-effectiveness across global healthcare systems.

## Data Availability

The datasets generated during and/or analyzed during the current study are available from the corresponding author on reasonable request.

## Abbreviations

*5-ALA*: 5-Aminolevulinic Acid
*BEV*: Bevacizumab
*CAN*: Canadian Dollar
*CEA*: Cost Effectiveness Analysis
*CHF*: Swiss Franc
*CNY*: Chinese Yen
*CPI*: Consumer Price Index
*CR*: Cost Report
*DAM*: Decision Analytic Model
*DEALE*: Declining Exponential Approximation of Life Expectancies
*EUR*: Euro
*FGS*: Fluorescence Guided Surgery
*GBM*: Glioblastoma
*GTR*: Gross Total Resection
*HCRU*: Healthcare Resource Utilization
*ICER*: Incremental Cost Effectiveness Ratio
*ICUR*: Incremental Cost Utility Ratio
*IDH*: Isocitrate Dehydrogenase
*JY*: Japanese Yen
*LOM*: Lomustine
*LYG*: Life Year Gained
*MeSH*: Medical Subject Headings
*MGMT*: O6-methylguanine-DNA methyltransferase
*MM*: Markov Model
*NA*: Not Available
*NGS*: Next Generation Sequencing
*NHB*: Net Health Benefit
*NMB*: Net Monetary Benefit
*OS*: Overall Survival
*PFS*: Progression Free Survival
*PICO*: Population/Patient, Intervention, Comparison, Outcome
*PPP*: Purchasing Power Parity
*PPPM*: Per Patient Per Month
*PRISMA*: Preferred Reporting Items for Systematic Reviews and Meta-Analyses
*PSM*: Partitioned Survival Model
*QALM*: Quality Adjusted Life Month
*QALY*: Quality Adjusted Life Year
*RT*: Radiotherapy
*TMZ*: Temozolomide
*TTF*: Tumor Treating Fields
*USA*: United States of America
*USD*: US Dollar
*WHO*: World Health Organization
*WTP*: Willingness To Pay
